# Leveraging biochemical reactions to unravel functional impacts of cancer somatic variants affecting protein interaction interfaces

**DOI:** 10.12688/f1000research.74395.2

**Published:** 2022-11-30

**Authors:** Francesco Raimondi, Joshua G. Burkhart, Matthew J. Betts, Robert B. Russell, Guanming Wu

**Affiliations:** 1 BIO@SNS, Scuola Normale Superiore di Pisa, Pisa, Italy, Italy; 2 Division of Bioinformatics and Computational Biology, Department of Medical Informatics and Clinical Epidemiology, Oregon Health & Science University, Portland, Oregon, USA; 3 Heidelberg University Biochemistry Center, University of Heidelberg, Heidelberg, Germany; 4 BioQuant, University of Heidelberg, Heidelberg, Germany

**Keywords:** Structural bioinformatics, network bioinformatics, biological process, variant interpretation, biochemical reactions, PPI network, biological pathways, cancer mutations, Reactome

## Abstract

**Background:** Considering protein mutations in their biological context is essential for understanding their functional impact, interpretation of high-dimensional datasets and development of effective targeted therapies in personalized medicine.

**Methods:** We combined the curated knowledge of biochemical reactions from Reactome with the analysis of interaction-mediating 3D interfaces from Mechismo. In addition, we provided a software tool for users to explore and browse the analysis results in a multi-scale perspective starting from pathways and reactions to protein-protein interactions and protein 3D structures.

**Results:** We analyzed somatic mutations from TCGA, revealing several significantly impacted reactions and pathways in specific cancer types. We found examples of genes not yet listed as oncodrivers, whose rare mutations were predicted to affect cancer processes similarly to known oncodrivers. Some identified processes lack any known oncodrivers, which suggests potentially new cancer-related processes (e.g. complement cascade reactions). Furthermore, we found that mutations perturbing certain processes are significantly associated with distinct phenotypes (i.e. survival time) in specific cancer types (e.g. PIK3CA centered pathways in LGG and UCEC cancer types), suggesting the translational potential of our approach for patient stratification. Our analysis also uncovered several druggable processes (e.g. GPCR signalling pathways) containing enriched reactions, providing support for new off-label therapeutic options.

**Conclusions: **In summary, we have established a multi-scale approach to study genetic variants based on protein-protein interaction 3D structures. Our approach is different from previously published studies in its focus on biochemical reactions and can be applied to other data types (e.g. post-translational modifications) collected for many types of disease.

## Introduction

Somatic mutations in cancer driving genes are a primary cause of oncogenesis. Large-scale cancer sequencing projects, including TCGA and ICGC, have uncovered many somatic mutations in cancer related genes. ^1^^,^^2^ Though a plethora of studies have been published investigating the molecular mechanisms of mutations in these genes and how they cause cancer, detailed and comprehensive biochemical mechanisms remain undiscovered. While recent efforts have been devoted to development of integrated bioinformatics pipelines for the reliable prediction of cancer driver mutation, ^3^ the mechanistic details from such predictions are usually not available, therefore hampering deeper biological interpretation and clinical translation. ^4^ The majority of current approaches for these studies are at the gene or protein, protein-protein interaction or pathway levels. The gene-level analysis tells us what changes (e.g. structural variants) occur in genes or proteins, but not the functional impact (e.g. biological processes), while the pathway-level analysis may reveal potential overall impact of mutations in cancer drivers, but not how. The protein-protein interaction level analysis may help us infer possible impact on the interactions of mutated proteins, but such analysis does not extend to the pathway context because a protein-protein interaction may be involved in multiple pathways. For example, an interaction between GAB1 and EGFR has been annotated by BioGrid ( https://thebiogrid.org/108824/summary/homo-sapiens/gab1.html) and involved in both Signaling by EGFR ( https://reactome.org/PathwayBrowser/#/R-HSA-177929) and Signaling by ERBB2 ( https://reactome.org/PathwayBrowser/#/R-HSA-1227986) as annotated in Reactome via different reactions.

Several studies have been published to survey cancer somatic variants using protein-protein interaction 3D structures (e.g. ^5^^–^^10^). However, these studies were all based on experimentally determined protein-protein 3D structures, which covers only 6% of currently known human protein-protein interactions based on the latest release of interactome3d ( https://interactome3d.irbbarcelona.org/results.php?queryid=human ^11^). To increase the coverage of 3D structures for protein-protein interactions, several groups (e.g. ^11^^–^^15^) have employed homology modeling approaches using 3D structures of homologous proteins as templates. Even using homology modeling, the structural coverage of the human interactome remains quite low (12% in interactome3d). The Mechismo approach ^16^ was developed to predict the functional consequences of variants and post-translational modifications (PTMs), such as phosphorylations and acetylations, at biomolecular 3D interfaces. The method extends predictions to proteins with no 3D structures as a function of sequence similarity to 3D templates assessed through BLAST alignments. ^17^ This tool allows for inspecting positional effects of mutations and modifications for protein-protein, protein-nucleic acids and a subset of protein-chemical interactions which we previously demonstrated to be as effective as comparative modeling in identifying residues at interaction interfaces without intensive computation.

Pathway and network-based approaches are routinely used in cancer data analysis. Projecting perturbed genes in the contexts of pathways and networks provides a knowledge framework for researchers to understand the potential roles of genetic aberrations that cause cancer. Pathway and network knowledgebases provide the foundation to perform pathway and network-based analysis. Reactome ^18^ is the most comprehensive open source biological pathway knowledgebase, covering nearly 60% of total human protein coding genes with annotation of over 13,200 complexes and 13,500 reactions that are organized into approximately 2,500 manually curated pathways (Release 75, December 2020). Pathways in Reactome are annotated as linked biochemical reactions, covering all types of biological processes, including metabolism, signal transduction, cell cycle, DNA replication and repair, programming cell death, immune system, development biology and cell-cell interaction.

Leveraging the protein-protein 3D structural information generated from Mechismo and the rich set of biochemical reactions in Reactome pathways, we conducted a systematic analysis of protein structural variants in cancers using the somatic mutation data from TCGA. Our analysis is focused on functional impact of variants at 3D interfaces mediating biochemical reactions, striking a balance between the gene or protein level and pathway level analyses and unraveling novel biochemical insights of oncogenesis. In addition, we have developed a new set of features for Reactome’s Cytoscape app, ReactomeFIViz, ^19^ enabling researchers to browse and explore results from this study.

## Results

### A multi-scale analysis approach for protein structural variants caused by cancer somatic mutations

**Figure 1.**
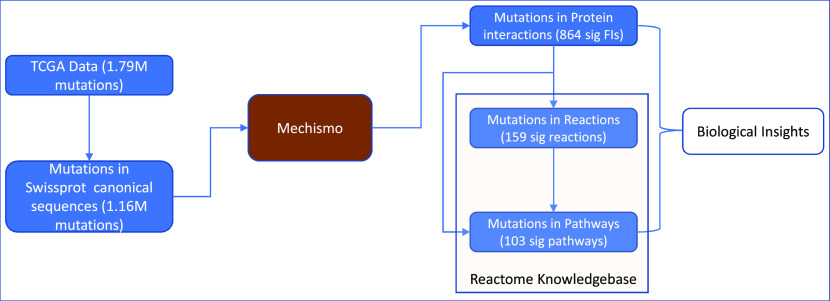
An overview of a multi-scale analysis approach for protein structural variants caused by cancer somatic mutations collected in the TCGA dataset.

We developed a robust analysis approach to analyze cancer somatic mutations in the context of protein-protein interactions, biological reactions, and pathways ( Figure 1). We applied this approach to the TCGA dataset by first mapping somatic mutations to UniProt canonical protein sequences and then onto 3D structures of protein-protein interactions as we have done previously through Mechismo. ^20^ The mapped protein-protein interactions are then traced back to reactions annotated in Reactome, which are tested for statistical significance. Significant pathways can be detected through similar statistical analysis (Methods) or may be found by mapping significant reactions to pathways that contain them. By combining the analysis results from protein-protein interactions, reactions and pathways, we draw biological insights.

### Mapping somatic mutations from genes to interaction interfaces using 3D structures unravels cancer driving mutations

**Figure 2.**
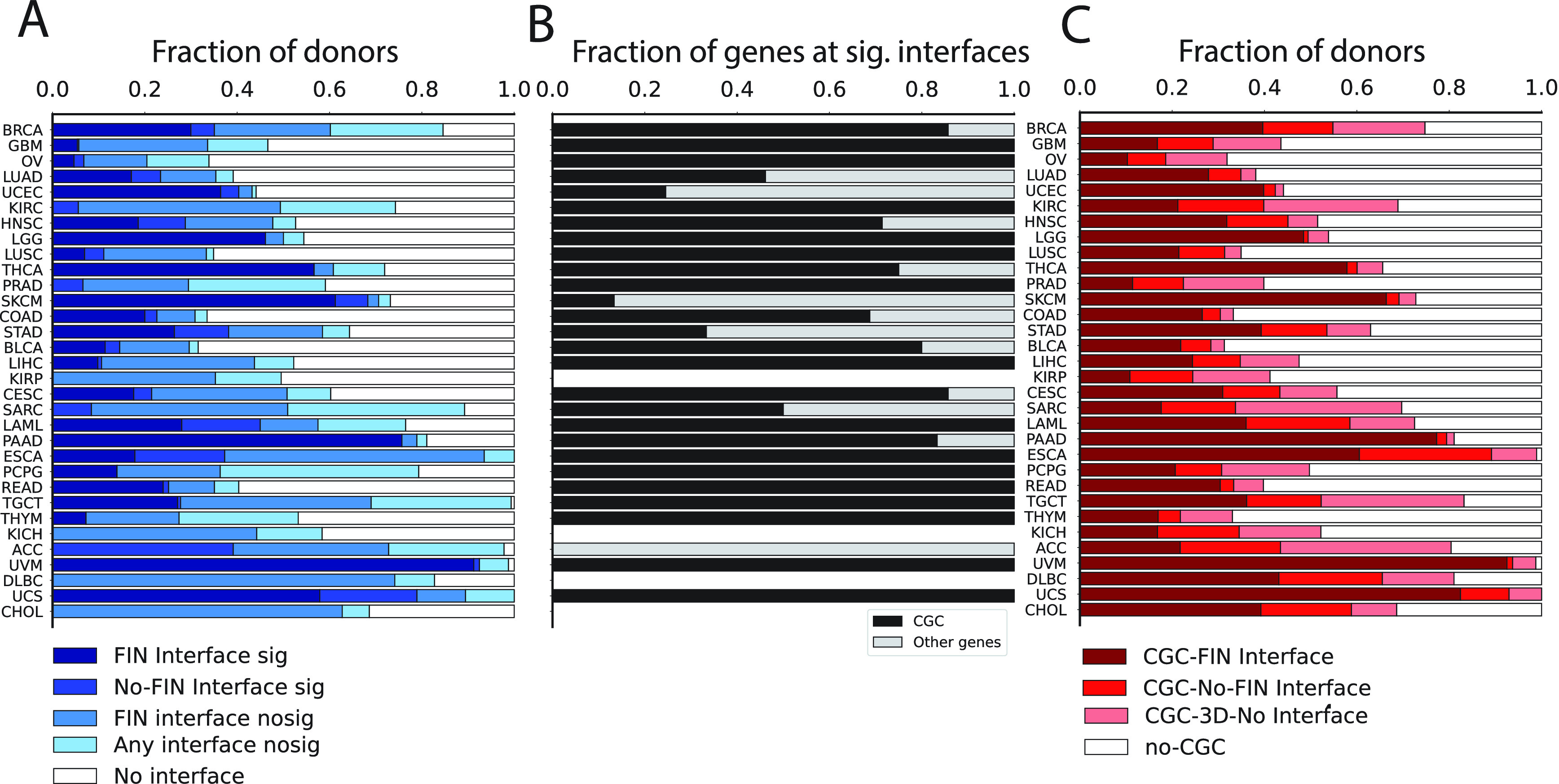
Statistics of the Mechismo analysis related to A) Significantly enriched interfaces; B) CGC gene fractions at significant interfaces from A); C) Structural mapping of CGC variants with no significance enrichment threshold applied. Cancers shown in rows were sorted based on total number of samples.

We considered a total of 1.79 M non-synonymous somatic mutations from TCGA across 32 cancer types and 7120 unique samples carrying somatic mutations. These were mapped onto canonical UniProt sequences, yielding 1.16 M unique protein variants (Methods). We considered high confidence predictions for protein-protein interactions including known structures or close (≥70% sequence identity) homologs and representing only very confident, physical protein-protein interactions. For chemical and DNA/RNA we also considered predictions with low/medium confidence (as low as 30% sequence identity). Mechismo analysis revealed that 405 K mutations (22%) mapped to known 3D structures, including single structural matches (i.e. with blast E-value threshold of 0.0001). A total of 103 K (25%) of them were found at interaction interfaces, for a total of 6668 (59%) samples carrying at least one interface perturbing mutation ( Figure 2A). The majority of mutations were found at protein-protein and protein-chemical interfaces (60 K and 48 K, respectively) while 14 K were predicted at protein-nucleic acid interfaces.

To define the biological processes affected by interface mutations, we mapped 3D interaction interfaces to Reactome’s Functional Interaction Network (FIN), ^21^ involving both protein-protein and protein-chemicals interactions (Methods). We matched 6559 of 17225 (38%) FIN Protein-chemical interactions and 24363 of 183672 (13%) FIN PPIs to 3D interaction interfaces. We assessed the statistical significance of mutation enrichment at interaction interfaces through a random background model obtained by shuffling the same substitutions between equivalent positions in the same proteins ( *Extended data:* Table S1 ^38^; Methods). Of the 2974 (26%) samples affected by significantly perturbed interfaces, 82% of them (2437 samples) carried mutations at significant FIN interfaces and 32% (3694 samples) carried mutations mapping either to non-significant interfaces or structural, non-interface regions. A total of 41% of samples (4705) carried mutations that couldn’t be mapped to any available 3D interaction interface ( Figure 2A).

The analysis revealed wide differences among cancer types in terms of fraction of samples affected by interface mutations. Five cancer types displayed more than 50% of samples carrying significantly impacted Functional Interactions (FIs): UVM (91%), PAAD (75%), SKCM (61%), UCS (57%) and THCA (56%) ( Figure 2A). Other cancer types displayed a higher portion of samples affected by mutations at significantly perturbed interfaces which cannot yet be mapped to Reactome processes (e.g. ACC). Finally, cancer types like KIRP, KICH, DLBC and CHOL displayed no samples significantly affected by mutations perturbing any known protein interaction interfaces ( Figure 2A).

More than half of cancer types with significantly affected interaction interfaces (15 out of 28) display only mutations at Cancer Genes Census (CGC) genes ^22^ at FI interfaces ( Figure 2B). After loosening the thresholds for enrichment significance, i.e. no False Discovery Rate (FDR) cutoff, we found a total of 3638 (32%) samples carried CGC gene mutations at FI interfaces ( Figure 2C). Cancer types with higher proportions of significantly affected FI interfaces also display greater contributions of CGC gene mutations at FI interfaces (e.g. UVM = 92%, UCS = 82%, PAAD = 77% and SKCM = 66%). Interestingly, we were able to detect patterns of CGC gene mutations at FI interfaces even in those cancer types with no significantly affected interaction interfaces (e.g. KIRP = 11%, CHOL = 39%, KICH = 17%, DLBC = 43%) ( Figure 2C).

### Placing mutations in the context of biochemical reactions and pathways reveals molecular mechanisms of cancer

We also assessed whether biological processes, via either individual reactions or whole pathways, were significantly affected by somatic mutations in terms of mediating interaction interfaces. Defining biological processes significantly affected by edgetic mutations, ^23^ instead of individual genes, allowed us to provide a mechanistic interpretation framework for low frequency mutations participating in common biological processes, thus facilitating the understanding of the oncogenetic mechanisms of cancer driver mutations in the long tail distribution. ^24^ A total of 25 cancer types displayed 556 significantly mutated reactions (corresponding to 460 interfaces and 150 genes) and 375 pathways (corresponding to 2397 interfaces and 759 genes) (see *Extended data:* Tables S2 and S3 ^38^). A total of 303 reactions displayed at least two genes affected by interface mutations and 20 reactions were found to be affected in at least five genes (Table S2). The reaction “MAP 2Ks and MAPKs bind to the activated RAF complex” in SKCM is the single reaction carrying the largest number of interface mutations (from 179 unique samples; Table S2) affecting participating members (i.e. *SRC, MAPK3, NRAS, RAP1A, MAP 2K2, ARAF, BRAF, KSR1, KSR2, RAF1*).

**Figure 3.**
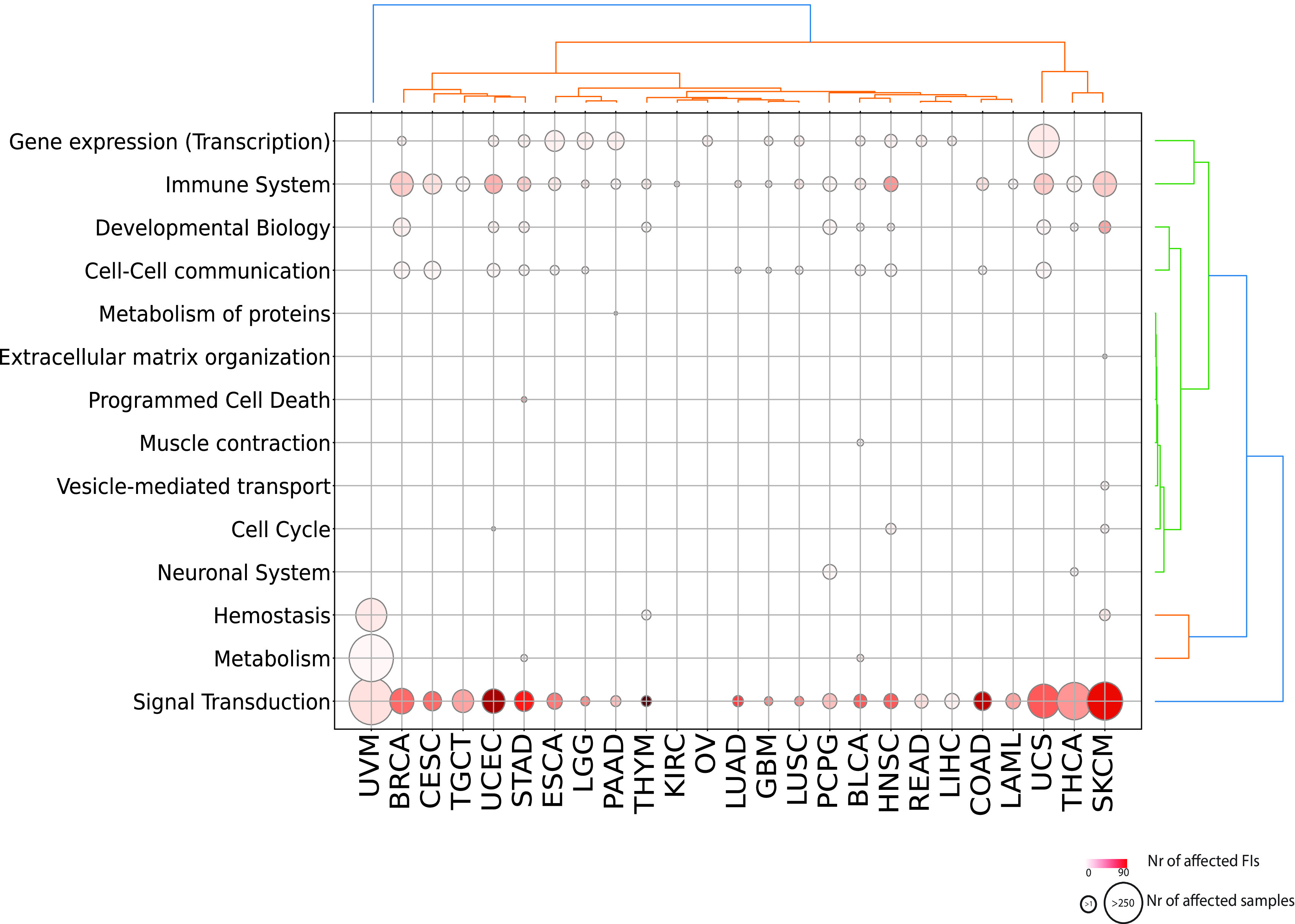
Significantly affected reactions in individual TCGA cancer types mapped to top level Reactome pathways. Diameters are proportional to the numbers of unique samples having mutations in top level pathways and color shades are proportional to the numbers of FI interfaces.

**Figure 4.**
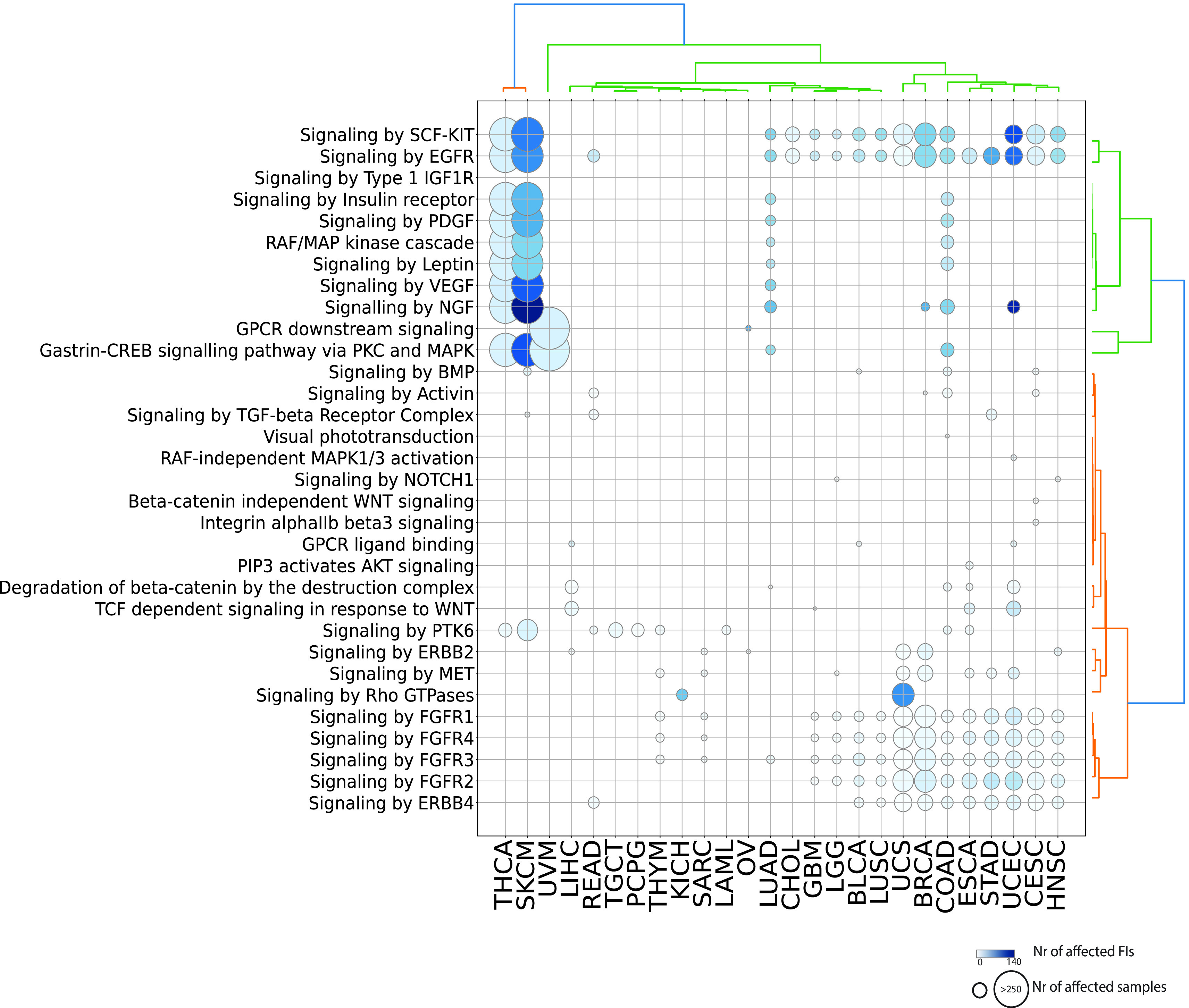
Significantly affected pathways belonging to the Signal Transduction domain. Diameters are proportional to the numbers of unique samples and color shades are proportional to the numbers of FI interfaces.

We obtained a landscape of cancer type specific patterns of perturbed processes by mapping each reaction or pathway to its top level biological pathway ( Figure 3; *Extended data:* Figure S1 ^38^). Signal transduction is the single most affected top level pathway, showing significantly perturbed reactions and pathways in 92% of cancer types (23 out of 25 and 26 out of 28 total cancer types displaying respectively significant reactions and pathways). Signal transduction processes also display high variability in terms of samples mutated and interfaces involved with melanoma (SKCM) involving the highest number of perturbed interfaces when considering either reactions or pathways ( Figure 3; Figure S1; firebrick). Given the high number of reactions affected in signaling events, it is easier to visualize significantly affected pathways ( Figure 4). As expected, receptor and non-receptor tyrosine kinase pathways are the most widely perturbed signaling pathways. In total, 10 out of 12 pathways from the “Signaling by Receptor Tyrosine Kinases” super-pathway ( https://reactome.org/PathwayBrowser/#/R-HSA-9006934&PATH=R-HSA-162582) are affected through cancer-type specific mutation signatures. *Signaling by EGFR* is the most recurrently perturbed pathway, being affected in 65% (17 out of 26) cancer types. Several other signaling pathways are related to GPCR signaling either through canonical pathways (i.e. *GPCR downstream signaling, Gastrin-CREB signaling pathway via PKC and MAPK, GPCR ligand, Signaling by Rho GTPases or PIP3 activates AKT signaling*) or through WNT signaling (i.e. *Beta-catenin independent WNT signaling, Degradation of beta-catenin by the destruction complex, TCF dependent signaling in response to WNT*).

Other top level processes that are widely perturbed are *Immune System* and *Gene Expression (Transcription)* ( *Extended data:* Figure S2 ^38^). Perturbed reactions linking to *Gene expression (Transcription)* are dominated exclusively by *TP53* reactions, which are affected in all the 14 cancer types, with the sole exception of UCS where an additional reaction is affected by *PPP2R1A* mutations (Figure S2). We found at least one reaction contributing to the *Immune System* process significantly affected in 84% (21 out of 25) of cancer types, for a total of 19 unique reactions. The majority of significantly perturbed reactions in Immune System (12 out of 19) appear selectively mutated in only one cancer type, suggesting shared steps of dysregulation of a key cancer process ( *Extended data:* Figure S3 ^38^).

**Figure 5.**
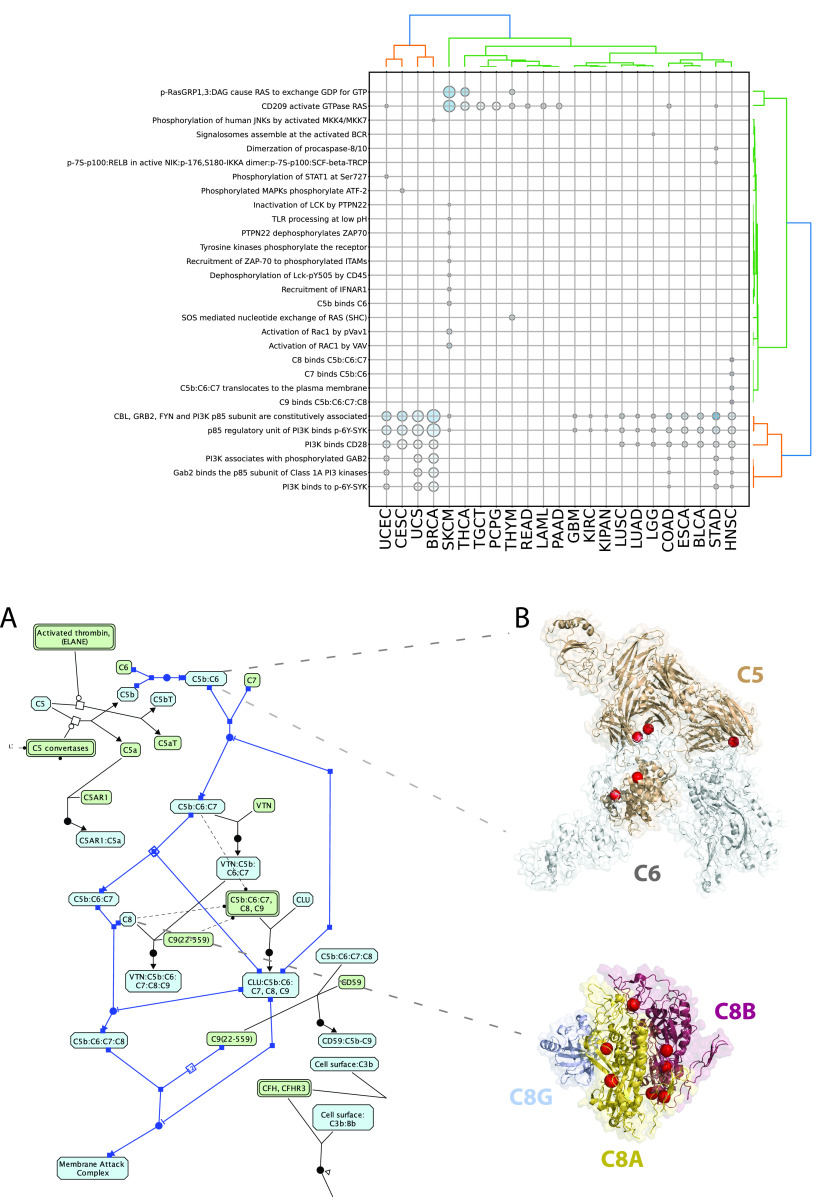
A) Reaction view of the complement cascade pathway; B) non-synonymous mutations (red spheres) at interfaces forming C5C6 complex (PDB:4a5w), participating to the “C5b binds C6” reaction and the C8 complex (PDB: 3ojy), participating to the “C7 binds C5b:C6”, “C8 binds C5b:C6:C7”, “C5b:C6:C7 translocates to the plasma membrane” and “C9 binds C5b:C6:C7:C8” reactions.

Intriguingly, we also found that a total of 15 significant reactions and 29 significant pathways in 8 and 14 cancer types, respectively, displayed no known CGC cancer drivers among the affected genes and were mutated in 10 or more unique samples in some cases (see Table S2 and S3). For example, we found complement cascade reactions, e.g. “C5b:C6:C7 translocates to the plasma membrane” ( Figure 5A), significantly perturbed in 12 HNSCC samples (FDR = 2.8·10 ^−3^) through mutations affecting five genes (i.e. *C8A*, *C9*, *C6*, *C8B*, *C8G*), which are predicted to disable the interaction with binding partners ( Figure 5B). Another reaction of the terminal pathway of complement, “C5b binds C6” ( Figure 5A), was found mutated in 11 SKCM samples (FDR = 1.2·10 ^−2^) at *C5* mediated interfaces, likewise predicted to perturb the interaction with binding partners ( Figure 5B). The complement cascade pathway is also overall significantly affected in HNSCC in 15 samples (FDR = 0.01) with no CGC gene mutations reported (Table S3). Additionally, a few other pathways are also affected in multiple cancer types with no mutations in CGC genes, including: Asparagine N-linked glycosylation (STAD FDR = 0.01, KIRC FDR = 0.02), ER to Golgi Anterograde Transport (STAD FDR = 0.02, KIRC FDR = 0.02), GPCR ligand binding (LIHC FDR = 0.003, BLCA FDR = 0.001) and Cilium Assembly (SKCM FDR = 0.03, LUAD FDR = 0.04) (Table S3).

### A cancer reaction network highlights cancer specific patterns and supports crosstalk among high level processes

**Figure 6.**
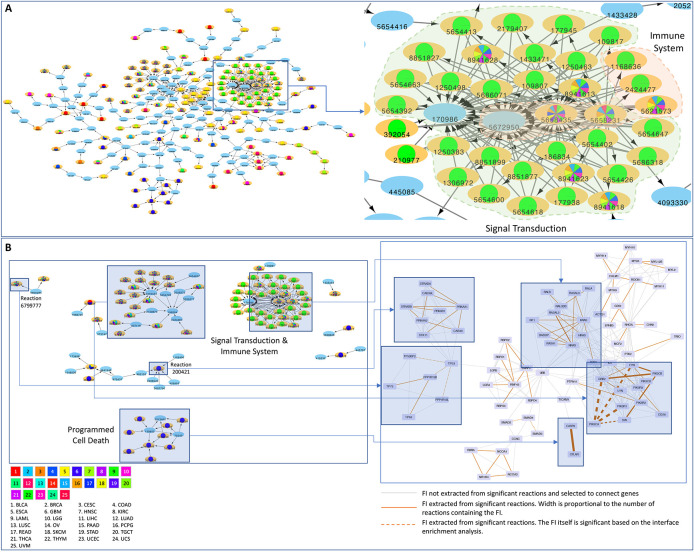
A) A Reactome reaction sub-network constructed based on significant reactions collected from cancer specific analysis. Reactions in light blue that are not significant are used as linker nodes to link significant reactions together. Cancer types where reactions are significant are shown as pie charts inside reaction nodes with different colors; B) The mapping between the significant reactions and functional interactions of STAD in both the reaction network and the FIN highlighting the advantage of survey protein variants in cancer with multi-scale perspectives.

Significantly affected reactions can be visualized on a reaction network, where nodes represent reactions and edges represent temporal or preceding/following relationships between reactions. Certain network modules are affected in specific cancer types such as STAD (e.g. Figure 6A, lower blue module; *Extended data*: Figure_6_Networks.cys) while others are widely perturbed in multiple tumor contexts. The network view also allows for enhanced visualization of reaction interdependencies. For example, we identified a major cluster suggesting crosstalk between Signal Transduction and Immune System reactions in multiple cancer types (the zoomed in region in Figure 6A). The majority of reactions in this cluster are significant in THYM. However, eight of them are significant in multiple cancer types. Reactions within the cluster are largely annotated for signaling transduction but five of them are annotated for immune system. In addition, reactions involving *PIK3CA* and *NRAS* (or *KRAS*), which are recurrently mutated in a mutually exclusive fashion in multiple cancer types, also support a major crosstalk between Signal Transduction and Immune Systems ( *Extended data:* Figures S3 and S4 ^38^).

The analysis pipeline we developed was used to survey the protein variants in cancer with a multi-scale perspective, allowing us to uncover cancer driver mutations significantly related to protein interactions or biochemical reactions and providing complementary results to help us draw biological insights. A reaction may be expanded into a set of functional interactions while a functional interaction may be involved in multiple reactions. Using STAD as an example ( Figure 6B; *Extended data*: Figure_6_Networks.cys), Reaction 679977 (TP53 family members bind PPP1R13B or TP53BP2, https://reactome.org/PathwayBrowser/#/R-HSA-6799777) can be expanded into four functional interactions (PPP1R13B-TP63, PPP1R13B-TP73, PPP1R13B-TP53 and TP53-TP53BP2), two of which (PPP1R13B-TP53 and TP53-TP53BP2) are statistically significant after FDR correction. Conversely, the interaction between *CASP8* and *CFLAR* is extracted from nine significant reactions that are annotated for programmed cell death ( https://reactome.org/PathwayBrowser/#/R-HSA-5357801). Interestingly, this interaction itself is not significant, presumably caused by FDR correction because there were many more protein interactions subject to analysis than reactions. Other interesting examples can be found for STAD in Figure 6B too. For example, Reaction 200421 (activation of cytosolic AMPK by phosphorylation, https://reactome.org/PathwayBrowser/#/R-HSA-200421), which is significant, is mapped to nine FIs that are not significant. The functional interaction between *PIK3CA* and *PIK3R1* is involved in 10 reactions and both the FI and all 10 reactions are significant.

### Linking mutations to phenotypes through biochemical mechanisms

To explore the translational potential of our approach, we checked whether patients carrying mutations perturbing either individual interfaces, reactions or pathways were found to be statistically associated with patient overall survival. When considering interfaces, we found only one instance and no significant interfaces involving *PIK3CA* ( *Extended data:* Table S4 ^38^). Grouping interface perturbing mutations through reactions or pathways increases the statistical power of survival analysis ( *Extended data:* Tables S5 and S6 ^38^). Indeed, we found six and ten significant pathways in LGG (Brain Lower Grade Glioma) and UCEC (Uterine Corpus Endometrial Carcinoma) (adj P (Cox) < 0.05) respectively, invariably involving *PIK3CA* along with different combinations of other oncogenes (e.g. *NRAS*, *KRAS*) interfaces. Intriguingly, in both LGG and UCEC *Signaling by Interleukins* pathway has the largest numbers of affected patients (respectively 27 and 105 samples; Table S6). LGG patients with mutations in this pathway exhibit shorter survival (hazard ratio = 2.08, adjusted p-value = 0.05) while UCEC patients show longer survival (hazard ratio = 0.35, adjusted p-value = 0.02), suggesting that patients carrying mutations of genes involved in the same pathways of recurrently mutated oncodrivers might cause different phenotypes (e.g. survival time) in different cancer contexts.

### Visualizing cancer somatic mutations via a multi-scale perspective using ReactomeFIViz

**Figure 7.**
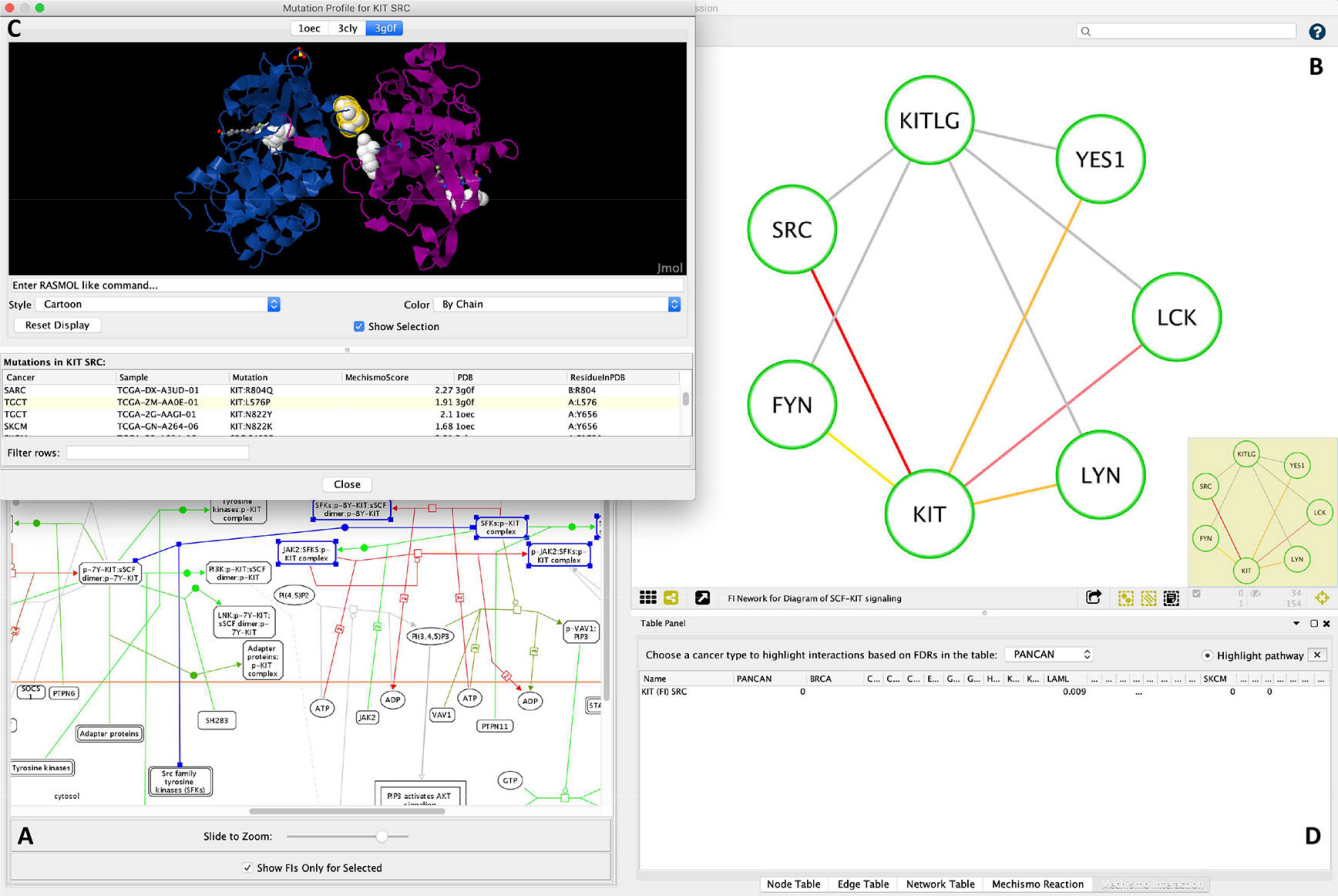
Visualization of structural variants in the context of Reactome pathways, reactions and functional interactions. A. Pathway diagram with reactions highlighted according to p-values from the structural analysis; B. Functional interactions for the selected reaction in A (in blue) with interactions highlighted according to p-values; C. Protein-protein interaction 3D view with mutation locations displayed in balls; D. Table control for users to choose a specific cancer to visualize its analysis results.

ReactomeFIViz ^19^ is Reactome’s Cytoscape app, implementing a suite of features for users to conduct pathway- and network-based data analysis and visualization based on Reactome pathways and its functional interaction network. To assist users to explore and browse the analysis results from this study, we implemented a set of new features in ReactomeFIViz. Figure 7 illustrates three views, allowing users to visualize variants in a multi-scale perspective ranging from pathways and reactions to protein-protein interactions and protein 3D structures. Users can load analysis results in the pathway diagram view to highlight reactions based on adjusted p-values ( Figure 7A) and select a specific reaction to inspect functional interactions impacted by mutations and highlighted according to adjusted p-values ( Figure 7B). Furthermore, users can also select a specific interaction and visualize mutation locations in the protein 3D structures pulled directly from PDB ( Figure 7C). ReactomeFIViz provides results from all TCGA cancers as well as pancancer, from which users can choose one to explore ( Figure 7D). For more information on how to use ReactomeFIViz to explore the analysis results, see the user guide at https://reactome.org/userguide/reactome-fiviz#Structural_Variants_Visualization.

## Discussion

In this study, we conduct a systematic analysis of protein variants in cancer by mapping non-synonymous cancer somatic mutations from TCGA to protein interaction interfaces using Mechismo and the Reactome functional interaction network first, and then mapping those interfaces back into biochemical reactions and pathways to search for statistically significant interactions, reactions and pathways. Mechismo offers the possibility to predict positional effects of mutations and PTMs at biomolecular interfaces without the need to homology model 3D complexes. Reactome is unique in providing a comprehensive set of manually annotated biochemical reactions which can be leveraged to interpret cancer somatic variants in the context of biochemical reactions and biological pathways. Our analysis is focused on biochemical reactions carried out by protein-protein and protein-chemical interactions and it differs from previous published studies, which were focused either on protein-protein interactions or biological pathways, e.g. ^25^ By focusing on biochemical reactions, we identified and provided mechanistic explanations for rare mutations impinging on reactions participating in established onco-pathways involving recurrently mutated oncodrivers (e.g. Receptor Tyrosine Kinase signaling). Our multiscale representation of biomolecular processes was instrumental in highlighting interactions or reactions playing roles in crosstalk between higher level pathways belonging to distinct domains of biological function (e.g. Signal Transduction and Immune Systems). We also identified significantly affected processes not involving any CGC, thereby representing potentially new cancer-associated processes. For example, we identified several interface mutations affecting the complement cascade pathway, which is emerging as a key regulator of the tumor microenvironment fostering tumor growth and metastasis. ^26^ Several of the identified pathways also represent new therapeutic opportunities readily targetable by off-label drugs. For instance, we identified several GPCR signaling pathways characterized by rare mutations on either the receptors or downstream signaling nodes, confirming our previous findings that sparse GPCR mutations might converge in dysregulating signaling cascades similar to recurrent mutations of coupled G-proteins. ^27^^,^^28^

On top of this study, we have implemented a set of new features in ReactomeFIViz for the community to browse and explore the results of our analysis, facilitating the understanding of potential cancer driver mechanisms in a unique, multi-scale perspective that is built upon the Reactome pathway knowledgebase and extends from gene and protein to protein interactions, reactions and pathways. Together with ReactomeFIViz’s previously existing features for visualizing drug-target interactions in the same context of pathways and interaction networks and fuzzy logic simulation to model perturbation of drugs and somatic mutations on pathway activities, ^29^ users are able to conduct in-silico drug screening and modeling in search of potential therapies by targeting significantly impacted reactions and related functional interactions. We will explore the utility of leveraging ReactomeFIViz in this way.

The study reported in this paper was based on protein-protein and protein-chemical interaction 3D structures. The limited coverage of protein-protein interaction 3D structures still constrains our survey to a small portion of protein-protein interactions extracted from biochemical reactions and pathways annotated in Reactome. In the future, when results and tools from ongoing large-scale protein structure determination ^30^ or prediction projects (e.g. CASP community ^31^) become available, we expect to obtain more insightful results by applying our approach.

In summary, we have developed a multi-scale approach to investigate protein structure variants in cancer in the context of protein interactions, reactions and pathways annotated in Reactome. This approach is generic and can be applied to study other types of abnormal protein omics features collected from cancer or other complex diseases. For example, abnormal up or down protein or gene expression data or post-translational modifications can be mapped to protein interactions, reactions, and pathways to investigate their potential impact. Our approach can also be easily extended to integrate other types of interactions, such as protein-DNA, protein-RNA and protein-membrane interactions, for which Reactome has annotated many complexes and reactions. We plan to conduct a more complete analysis by including all these interactions as well as integrated mutations and PTMs sets (e.g. from CPTAC proteogenomics studies) in the future.

## Methods

### TCGA dataset

We downloaded the latest MAF files of 32 TCGA Cancer types from http://firebrowse.org/ (2016 release), for a total of 1.8M somatic mutations from 7120 patients. A total of 1.79M non-synonymous somatic mutations were mapped on canonical UniProt sequences, yielding 1.16M unique protein variants. Only Ensembl transcripts matching in length to corresponding UniProt sequences were retained using Oncotator ^32^ “UniProt Exact Match” function and only predicted variants whose reference amino acids matched those at corresponding positions in SwissProt sequences were retained for further inspection. We employed standard TCGA study abbreviations (defined here: https://gdc.cancer.gov/resources-tcga-users/tcga-code-tables/tcga-study-abbreviations) for cancer type names.

### Constructing the Reactome FI network and the reaction network

We adapted the algorithm we developed to build the Reactome FI network ^21^ to extract functional interactions between proteins and proteins or proteins and chemicals from Reactome annotated reactions. The FI network used in this study is different from our previously constructed FI network. Here we limited our analyses to FIs extracted from annotated Reactome reactions only. To construct the Reactome reaction network, we connect two reactions (e.g. reaction1 and reaction2) together if the output from reaction1 is an input, catalyst, or regulator of reaction2. To control spurious connections among reactions, a set of small molecules, including ATP, ADP, Pi, H2O, GTP, GDP, CO2 and H+, is excluded for this reaction connection checking. The version of the Reactome knowledgebase used in this study is release 59, released in December 2016, which may have some annotations that don’t exist in the current Reactome release.

### Mapping Reactome FIs to Mechismo interfaces

We mapped protein-protein and protein-chemical functional interactions from Reactome FIN to 3D interaction interfaces from Mechismo ( mechismo.russelllab.org). ^16^ We used UniProt accessions to map protein entities and employed a chemoinformatics similarity search to map chemical entities. In more detail, we retrieved InChI identifiers for both ChEBI ^33^ (cross-referenced to Reactome) and PDB ^34^ (cross-referenced to Mechismo) chemical components. We then generated topological fingerprints for both sets through the RDKit package ( https://www.rdkit.org/) using the Tanimoto score as a similarity metric. We considered only interactions with matching UniProt accessions and with chemical similarity greater than 0.5 as corresponding protein-chemicals interactions.

### Using Mechismo to define perturbed FI interfaces

We predicted functional consequences of TCGA non-synonymous somatic mutations using Mechismo, ^16^ which matches protein positions to positions within structures and identifies sites affecting interactions with other proteins, DNA/RNA or small-molecules. UniProt sequences were aligned to PDB sequences via BLAST ^17^ considering matches with E-value threshold of 0.0001 and storing the best match for each position in the UniProt sequence. We considered high confidence predictions for protein-protein interactions including known structures or close (≥70% sequence identity) homologs and representing only very confident, physical protein-protein interactions. For chemical and DNA/RNA we also considered predictions with low/medium confidence (as low as 30% sequence identity).

We identified the most perturbed interactions in cancer by ranking each interacting pair based on the number of unique samples where a missense mutation was predicted to affect the interface. We tested the significance of the most perturbed interactions in TCGA by using an interactome perturbation random background model (BM). We defined it by first collecting the missense variants found in each sample and randomly shuffling the same substitutions between equivalent positions in the same protein. We obtained equivalent Mechismo data for BM and calculated the probability of obtaining the same number of observed perturbing events for each interaction by chance using a binomial test:

$$P\left(c,N\right)=\left(\begin{array}{c} N\\ c \end{array}\right){\left(P_{r}\right)}^{c}{\left(1-P_{r}\right)}^{N-c}$$

where *N* is the total number of samples, *c* is the number of unique samples in which a given interaction has been found perturbed and *P* is the probability of getting the same interface perturbed from the random background distribution. The obtained *P*-values were adjusted using the FDR/Benjamini-Hochberg procedure. ^35^ We considered instances with adjusted *P*-values below 0.05 as significant.

We similarly applied the same BM and statistical test to define reactions and pathways whose mediating FIs' interfaces are significantly affected.

### Survival analysis

We downloaded clinical records of TCGA patients using TCGAbiolinks Bioconductor package ^36^ and considered “vital_ status” , “gender”,”days_to_death”, “days_to_last_follow_up”, “age_at_diagnosis”. Cox’s proportional hazard model was employed to predict hazard ratios and survival probability of patients affected by interface-, reaction- and pathway-perturbing mutations, employing age and sex as covariates. Samples with missing values (i.e. “nan”) in any of these fields were not considered for this analysis.

All the statistical analysis has been done in Python 3.8.11 ( www.python.org) through scipy 1.6.2 ( www.scipy.org), statsmodels 0.12.2 ( statsmodels.sourceforge.net) and lifelines 0.25.9 ( lifelines.readthedocs.org/en/latest) libraries.

### Implementing new features in ReactomeFIViz

We developed a MySQL database to store the analysis results via a Hibernate API ( https://hibernate.org) and a RESTful API to serve the analysis results from the MySQL database to ReactomeFIViz using the Spring MVC framework (version 4.3.10, https://spring.io/projects/spring-framework). New user interfaces were added to ReactomeFIViz using Java Swing (Java 8, https://docs.oracle.com/javase/tutorial/uiswing/) by following the standard practice to develop apps for Cytoscape. ^37^ We used Java (JDK 1.8) for software programming and host the source code at GitHub: https://github.com/reactome-fi/mechismows for the RESTful API and https://github.com/reactome-fi/CytoscapePlugIn for the front-end user interfaces in ReactomeFIViz.

## Data availability

### Underlying data

MAF files of the 32 TCGA cancer types available from http://firebrowse.org/ (2016 release)

The outcome of the analysis is available through the Reactome FIViz Cytoscape app: https://reactome.org/tools/reactome-fiviz

The mysql database dump used for the server-side application: https://doi.org/10.5281/zenodo.5590953.

### Extended data

Zenodo: Leveraging biochemical reactions to unravel functional impacts of cancer somatic variants affecting protein interaction interfaces, https://doi.org/10.5281/zenodo.7358737. ^38^

This project contains the following extended data:

• Figure S1: Significantly affected pathways in individual TCGA cancer types mapped to top level Reactome pathways. Diameters are proportional to the numbers of unique samples having mutations in top level pathways and color shades are proportional to the numbers of FI interfaces.
• Figure S2: Significantly affected reactions belonging to the Gene expression top level pathway. Diameters are proportional to the numbers of unique samples and colors are proportional to the numbers of FI interfaces.
• Figure S3: Significantly affected reactions belonging to the Immune System top level pathway. Diameters are proportional to the numbers of unique samples and colors are proportional to the numbers of FI interfaces.
• Figure S4: Significantly affected reactions belonging to the Signal Transduction top level pathway. Diameters are proportional to the numbers of unique samples and colors are proportional to the numbers of FI interfaces.
• Figure_6_Networks.cys: The original Cytoscape session file containing networks shown in Figure 6. The file was generated using Cytoscape 3.8.2. The visual style of the functional interaction network in Figure 6B is different from the network labeled “Figure_6B_FIN” in this session file to better distinguish protein nodes from reaction nodes.
• Table S1: Mutation enrichment at protein interaction interfaces.
• Table S2: Mutation enrichment at Reactome Reactions interfaces.
• Table S3: Mutation enrichment at Reactome Pathway interfaces.
• Table S4: Survival analysis of significantly affected interactions.
• Table S5: Survival analysis of significantly affected reactions.
• Table S6: Survival analysis of significantly affected pathways.

Data are available under the terms of the Creative Commons Attribution 4.0 International license (CC-BY 4.0).

## Software availability

Home page for user guide describing procedures on how to use ReactomeFIViz features for visualizing cancer somatic mutations via a multi-scale perspective: https://reactome.org/userguide/reactome-fiviz#Structural_Variants_Visualization

Cytoscape app store: http://apps.cytoscape.org/apps/reactomefiplugin

Latest source code: https://github.com/reactome-fi/CytoscapePlugIn (ReactomeFIViz app code) and https://github.com/reactome-fi/mechismows (Server-side code)

Source code as at the time of publication: https://github.com/reactome-fi/CytoscapePlugIn/releases/tag/f1000_cancer_2021 and https://github.com/reactome-fi/mechismows/releases/tag/F1000_Cancer_2021

Archived source code as at the time of publication: https://doi.org/10.5281/zenodo.5590945 ^39^ (ReactomeFIViz app code) and https://doi.org/10.5281/zenodo.5590949 ^40^ (Server-side code)

License: Creative Commons Attribution 4.0 International (CC BY 4.0) License ( https://reactome.org/license).
