# Supplementary material for: Leveraging biochemical reactions to unravel functional impacts of cancer somatic variants affecting protein interaction interfaces
Source: F1000Res. 2022 Dec 12;10:1111. Originally published 2021 Nov 3. [Version 3] doi: 10.12688/f1000research.74395.3 (PMC9755755; doi:10.12688/f1000research.74395.3)
Supplement: Peer review history

# Peer review: Reviewer response for version 3

**DOI:** 10.5256/f1000research.141738.r157982

**Published:** 2022-12-15

**Authors:** Andras Szilagyi

**Affiliations:** 1 Institute of Enzymology, Research Centre for Natural Sciences, Budapest, Hungary

No new comments.

Is the work clearly and accurately presented and does it cite the current literature?

Yes

If applicable, is the statistical analysis and its interpretation appropriate?

Yes

Are all the source data underlying the results available to ensure full reproducibility?

Yes

Is the study design appropriate and is the work technically sound?

Yes

Are the conclusions drawn adequately supported by the results?

Yes

Are sufficient details of methods and analysis provided to allow replication by others?

Yes

Reviewer Expertise:

Structural bioinformatics

I confirm that I have read this submission and believe that I have an appropriate level of expertise to confirm that it is of an acceptable scientific standard.

# Peer review: Reviewer response for version 2

**DOI:** 10.5256/f1000research.141560.r156974

**Published:** 2022-11-30

**Authors:** Pier Luigi Martelli

**Affiliations:** 1 Biocomputing Group, Department of Pharmacy and Biotechnology, University of Bologna, Bologna, Italy

The present version addresses my concerns.

Is the work clearly and accurately presented and does it cite the current literature?

Yes

If applicable, is the statistical analysis and its interpretation appropriate?

Yes

Are all the source data underlying the results available to ensure full reproducibility?

Yes

Is the study design appropriate and is the work technically sound?

Yes

Are the conclusions drawn adequately supported by the results?

Yes

Are sufficient details of methods and analysis provided to allow replication by others?

Yes

Reviewer Expertise:

Computational biology; Bioinformatics; Structural and functional characterization of proteins and their variants; Machine and deep learning.

I confirm that I have read this submission and believe that I have an appropriate level of expertise to confirm that it is of an acceptable scientific standard.

# Peer review: Reviewer response for version 1

**DOI:** 10.5256/f1000research.78146.r147340

**Published:** 2022-09-20

**Authors:** Pier Luigi Martelli

**Affiliations:** 1 Biocomputing Group, Department of Pharmacy and Biotechnology, University of Bologna, Bologna, Italy

The paper by Raimondi et al. presents an analysis aiming at elucidating the effect of somatic, cancer-related variants on interaction interfaces of complexes involved in biochemical reactions. Data from 7120 patients of 32 cancer types are downloaded from TGCA and analysed with Mechismo for determining the possible functional effect of variations at protein-protein interfaces. Functional interfaces are then mapped on Reactome reactions and pathway and an enrichment procedure is adopted to highlight the most relevant biochemical processes involved in each cancer type.

 The paper is generally clearly written. In my opinion, paper readability would benefit from a more extended review of the methods at the basis of Mechismo and of the definition of functional interfaces. In particular, it is unclear what is the expected number of variations that Mechismo can annotate on proteins without 3D structure, as reported in the introduction. For the 1.16 M unique variations from TCGA, authors only report the number of those mapped on known 3D structures, and it is unclear whether they include those deriving from homology-based analysis. If yes, it would be useful to provide some more detail and discuss possible differences of effect predictions depending on the similarity between the wildtype sequence and the adopted structure.

 Other minor points:

 - Introduction: the sentence "The gene-level analysis tells us what changes occur in genes or proteins, but not the functional impact" is a bit misleading, since different approaches have been developed for predicting possible impact at different levels (function, stability...)

 - Legends of figures could be improved, adding details. In particular it is not clear what "donor" means in Figure 2.

 - Supplementary figures S1-S4 are unreadable in the current version.

Is the work clearly and accurately presented and does it cite the current literature?

Yes

If applicable, is the statistical analysis and its interpretation appropriate?

Yes

Are all the source data underlying the results available to ensure full reproducibility?

Yes

Is the study design appropriate and is the work technically sound?

Yes

Are the conclusions drawn adequately supported by the results?

Yes

Are sufficient details of methods and analysis provided to allow replication by others?

Yes

Reviewer Expertise:

Computational biology; Bioinformatics; Structural and functional characterization of proteins and their variants; Machine and deep learning.

I confirm that I have read this submission and believe that I have an appropriate level of expertise to confirm that it is of an acceptable scientific standard.

# Response

**Published:** 2022-11-25

**Authors:** Guanming Wu

**Affiliations:** Oregon Health & Science University, USA

Dear Dr. Pier Luigi Martelli,

 Thanks a lot for reviewing our manuscript. We have made some changes according to your comments. Please see our reply to your comments below:

- In my opinion, paper readability would benefit from a more extended review of the methods at the basis of Mechismo and of the definition of functional interfaces. In particular, it is unclear what is the expected number of variations that Mechismo can annotate on proteins without 3D structure, as reported in the introduction. For the 1.16 M unique variations from TCGA, authors only report the number of those mapped on known 3D structures, and it is unclear whether they include those deriving from homology-based analysis. If yes, it would be useful to provide some more detail and discuss possible differences of effect predictions depending on the similarity between the wildtype sequence and the adopted structure.

*We mapped mutations to 3D structures, either known or homologous, by considering UniProt alignment to PDB fasta sequences via BLAST, considering matches with E-value threshold of 0.0001, and storing the best match for each position in the UniProt sequence.*

*Then we considered high confidence predictions for protein-protein interactions including known structures or close (>=70% sequence identity) homologs and representing only very confident, physical protein-protein interactions. For chemical and DNA/RNA we also considered predictions with low/medium confidence (as low as 30% sequence identity). We have made these points clearer both in the Results and Methods sections.*

- Introduction: the sentence "The gene-level analysis tells us what changes occur in genes or proteins, but not the functional impact" is a bit misleading, since different approaches have been developed for predicting possible impact at different levels (function, stability...)

*When we said “gene-level analysis”, we meant the analysis is about genes or proteins such as somatic mutations or structural variants. Indeed, many approaches have developed to predict impacts of mutations on protein stability and functions. But we’d like to argue that “stability” is still a protein-level feature. To know the impact of mutation on the protein’s function, extra information is needed (e.g. what pathways or biological processes have been annotated for the protein, etc.) for higher level analysis (e.g. pathway enrichment analysis). Regardless, we added some phrases (as shown below in parentheses) to the original sentence. Now this sentence reads as the following:*

*The gene-level analysis tells us what changes (e.g. structural variants) occur in genes or proteins, but not the functional impact (e.g. biological processes)…*

- Legends of figures could be improved, adding details. In particular it is not clear what "donor" means in Figure 2.

*Thanks for pointing this out. We have changed “donors” to “patients” in Figure 2.*

- Supplementary figures S1-S4 are unreadable in the current version.

*These figures have been fixed. It is our mistake.*

 Thanks again for reviewing our manuscript and comments.

# Peer review: Reviewer response for version 1

**DOI:** 10.5256/f1000research.78146.r135297

**Published:** 2022-05-10

**Authors:** Andras Szilagyi

**Affiliations:** 1 Institute of Enzymology, Research Centre for Natural Sciences, Budapest, Hungary

In this impressive work, the authors investigated all available data on somatic cancer mutations by connecting 3D protein-protein and protein-chemical interface data with data on biochemical reactions and pathways, thereby revealing significantly impacted reactions and pathways. This novel approach has yielded numerous important insights. Analysis of patient survival times identified mutations perturbing processes affecting patient survival. The authors also created an updated visualization tool enabling the users to explore the results of the analyses.

 The methodology is sound, and the results support the conclusions. There are only a few technical problems which need to be fixed:

- The supplementary figures (Extended data) cannot be evaluated because they got cropped, probably during some conversion step.
- The top part of Fig. 5 is not labeled, and the legend does not refer to it.
- On Page 3, 'ERBB2' is misspelled as 'ERRB2'.
- The resolution of Fig. 6 is poor and most labels are unreadable. Since this figure is very complex, a vector image (preferably SVG) would be better. The legend says some nodes contain bar charts, but in fact they are pie charts. Actually, bar charts would be better because there are too many similar colors in the pie charts.
- The supplementary tables are provided in an Excel file which is inconvenient to use as the column labels are not fully visible. Please increase the height of the row for the column labels and use wrapped text.
- The cancer types are indicated with acronyms, but they are not expanded anywhere. Please provide their expansions, or a link where these can be found.

Regarding the survival analysis, the authors used the clinical records from TCGA. Were there any missing data? If yes, how were they handled?

 This article is a very significant contribution. However, it cannot be fully evaluated until the supplementary figures are fixed.

Is the work clearly and accurately presented and does it cite the current literature?

Yes

If applicable, is the statistical analysis and its interpretation appropriate?

Yes

Are all the source data underlying the results available to ensure full reproducibility?

Yes

Is the study design appropriate and is the work technically sound?

Yes

Are the conclusions drawn adequately supported by the results?

Yes

Are sufficient details of methods and analysis provided to allow replication by others?

Yes

Reviewer Expertise:

Bioinformatics, protein structure, protein-protein interactions, pathway analysis

I confirm that I have read this submission and believe that I have an appropriate level of expertise to confirm that it is of an acceptable scientific standard, however I have significant reservations, as outlined above.

# Response

**Published:** 2022-11-25

**Authors:** Guanming Wu

**Affiliations:** Oregon Health & Science University, USA

Dear Dr. Szilagyi,

 Thanks a lot for reviewing our manuscript. We have made the following updates according to your comments:

- The supplementary figures (Extended data) cannot be evaluated because they got cropped, probably during some conversion step.

*Our mistake. This has been fixed now.*

- The top part of Fig. 5 is not labeled, and the legend does not refer to it.

*The top part of Fig 5 is deleted.*

- On Page 3, 'ERBB2' is misspelled as 'ERRB2'.

*Thanks! Fixed.*

- The resolution of Fig. 6 is poor and most labels are unreadable. Since this figure is very complex, a vector image (preferably SVG) would be better. The legend says some nodes contain bar charts, but in fact they are pie charts. Actually, bar charts would be better because there are too many similar colors in the pie charts.

*Thanks for pointing this out. We replaced the figure with a PDF version, which is scalable. Furthermore, we provided the original Cytoscape file (Figure_6_Networks.cys) in Extended Data for readers to investigate the networks using Cytoscape, which can be downloaded from https://cytoscape.org/.*

*We tried to use bar charts in Cytoscape. However, we believe using pie charts is better. Also we fixed the name of the charts to “pie charts” from “bar charts”. Thanks a lot for your suggestion!*

- The supplementary tables are provided in an Excel file which is inconvenient to use as the column labels are not fully visible. Please increase the height of the row for the column labels and use wrapped text.

*Thanks for the suggestions. We have increased the height of the row for the column labels and use wrapped text.*

- The cancer types are indicated with acronyms, but they are not expanded anywhere. Please provide their expansions, or a link where these can be found.

*Regarding the cancer types acronyms, these are the standard abbreviations provided by TCGA ( https://gdc.cancer.gov/resources-tcga-users/tcga-code-tables/tcga-study-abbreviations). We provided a reference in the “TCGA dataset” section of the Methods.*

- Regarding the survival analysis, the authors used the clinical records from TCGA. Were there any missing data? If yes, how were they handled?

*Thanks for pointing this out. We didn’t consider in our analysis samples with missing values. We have now made this point clear in the “Survival analysis” section of the Methods.*

 Thanks again for your comments.
